# Sulfur fertilization optimizes maize yield through nutrient regulation and biomass responses in six contrasting soils of Northeast China

**DOI:** 10.3389/fpls.2026.1750011

**Published:** 2026-03-13

**Authors:** Shuai Cui, Shuoran Liu, Yu Zhang, Xinyuan Zhang, Qiang Gao

**Affiliations:** 1 Key Laboratory of Sustainable Utilization of Soil Resources in The Commodity Grain Bases of Jilin Province, College of Resource and Environmental Sciences, Jilin Agricultural University, Changchun, China; 2 School of Tropical Agriculture and Forestry, Hainan University, Haikou, China

**Keywords:** biomass, nitrogen, soil type, sulfur, yield, *Zea mays* L.

## Abstract

**Introduction:**

Sulfur (S) deficiency has re-emerged as a constraint to maize production due to reduced atmospheric deposition and the widespread use of S-free fertilizers. However, how S fertilization influences yield formation under contrasting soil conditions in Northeast China remains insufficiently quantified.

**Methods:**

Here, a two-year field experiment was conducted on six representative soil types using five S rates (0, 30, 60, 90, and 120 kg S ha^-^¹) with measurements of grain yield and its components, S and N accumulation and stoichiometry, and dry matter production and remobilization before and after silking.

**Results:**

Across the six soils, fitted optimal S rates (61.4–89.7 kg S ha^-^¹) increased grain yield by 2.5%-25.8%, with the largest responses observed in black soil and aeolian sandy soil. Yield gains were mainly associated with increases in kernel number and thousand-kernel weight, supported by path analysis. Moderate S supply (S60–S90) enhanced S and N uptake at silking and maturity, improved N/S ratios, and increased pre-silking biomass accumulation. Biomass remobilization efficiency increased from 5.0% (S0) to 11.6%–11.7% (S60–S90), contributing to a higher harvest index (40.8% at S0 vs. 42.6%–42.7% at S30–S90). Pre-silking biomass was positively related to grain yield across soils.

**Discussion:**

Overall, under the studied conditions, S fertilization improved maize yield primarily through promoting early-season growth, nutrient coordination, and biomass remobilization rather than increasing post-silking assimilation alone. These findings provide a scientific basis for soil-specific sulfur management in temperate spring maize systems of Northeast China.

## 1 Introduction

Sulfur (S) has re-emerged as a growth-limiting nutrient in many intensive cropping systems due to declining atmospheric sulfur deposition, reduced organic fertilizer inputs, and the widespread replacement of sulfate-containing fertilizers with highly concentrated phosphorus and nitrogen sources (Li et al., 2017; Schnug and Landwirtschaft, 2005). These changes have substantially altered soil sulfur supply capacity and increased the frequency of crop responses to sulfur fertilization. As an essential macronutrient, sulfur is indispensable for plant growth and development, playing critical roles in protein synthesis, enzyme activation, redox regulation, and nitrogen (N) metabolism. Recent syntheses further emphasize that sulfur nutrition contributes not only to alleviating deficiency symptoms but also to regulating yield stability and stress tolerance in modern agricultural systems (Narayan et al., 2023).

Beyond its direct physiological functions, sulfur nutrition is closely linked to the uptake, assimilation, and coordination of other mineral nutrients, particularly nitrogen. Sulfur availability affects nitrogen metabolism through its involvement in cysteine and methionine synthesis and influences whole-plant nutrient balance via coordinated biochemical and rhizosphere processes (Sharma et al., 2024). Agronomic evidence across diverse cropping systems indicates that sulfur fertilization can enhance nutrient uptake efficiency, promote biomass accumulation, and improve grain yield under field conditions (Mehmood et al., 2021). Collectively, these findings suggest that sulfur functions as an integral component of nutrient regulation networks rather than merely as a corrective input for visibly sulfur-deficient soils.

From an ecophysiological perspective, a foundational concept in maize production is the tight coupling between nutrient acquisition and biomass accumulation. Classical studies established that nutrient uptake dynamics are strongly associated with crop growth rate and that maintaining a critical nutrient concentration is required to sustain maximal biomass formation (Plénet and Lemaire, 1999; Guo et al., 2025). This nutrient–biomass framework has been repeatedly validated in maize, particularly under nitrogen-focused management, and provides a conceptual basis for understanding yield formation through coordinated regulation of nutrient uptake, canopy development, and assimilate partitioning (Liu et al., 2022a). Subsequent agronomic studies have shown that integrated optimization of nitrogen rate, planting density, and canopy structure enhances nutrient uptake, prolongs functional leaf area duration, increases pre- and post-silking biomass accumulation, and ultimately improves grain yield (Zhiipao et al., 2023; Balaout et al., 2025). However, the extent to which sulfur nutrition participates in or modifies this nutrient–biomass regulatory framework remains insufficiently explored.

Maize yield formation is particularly sensitive to assimilate supply during the critical period surrounding silking. Pre-silking nutrient uptake and biomass accumulation play decisive roles in ear differentiation, floret survival, and kernel set, making kernel number highly responsive to nutrient availability before silking (Zhai et al., 2022b, Zhai et al., 2022a). Nutrient-efficient genotypes and optimized nutrient and canopy management practices increase nutrient acquisition prior to silking, thereby supporting greater floret primordia development and higher kernel numbers (Liu et al., 2022b, Liu et al., 2022c). Although sulfur fertilization has been reported to enhance nutrient uptake, photosynthetic performance, biomass accumulation, and assimilate remobilization in cereals and legumes (Li et al., 2019), the specific pathways through which sulfur regulates pre-silking growth, biomass allocation, and subsequent assimilate remobilization to grain in maize remain poorly quantified, particularly under field conditions across soils with contrasting sulfur supply capacities.

In Northeast China, sulfur deficiency has intensified over recent decades due to decreasing atmospheric sulfur inputs, reduced organic amendments, and high crop nutrient removal rates. Jilin Province represents a major maize-producing region characterized by pronounced soil heterogeneity, encompassing black soils, aeolian sandy soils, saline–alkali soils, and other upland soil types with contrasting physicochemical properties and sulfur availability. Such heterogeneity provides a valuable natural framework for examining whether sulfur-mediated nutrient–biomass regulatory mechanisms are conserved across soils or are strongly modulated by soil-specific sulfur supply capacity. Despite increasing recognition of sulfur deficiency in this region, mechanistic understanding of how sulfur fertilization regulates maize yield formation across different soil types remains limited.

Two key knowledge gaps therefore persist: (i) how sulfur fertilization regulates maize yield, nutrient uptake, and biomass dynamics under field conditions across contrasting soil types; and (ii) whether sulfur-driven nutrient–biomass interactions represent a conserved regulatory mechanism or exhibit soil-dependent variability due to differences in inherent sulfur supply capacity. Addressing these gaps is essential for developing soil-targeted and efficient sulfur management strategies in temperate maize production systems. To address these questions, we conducted two-year field experiments across six representative upland soil types in Jilin Province, Northeast China, using a gradient of sulfur application rates. The objectives were to quantify the effects of sulfur fertilization on maize grain yield and its components, sulfur and nitrogen uptake dynamics, N/S stoichiometry, pre- and post-silking biomass accumulation and remobilization, and harvest index. Path analysis was applied to disentangle the direct and indirect contributions of nutrient uptake and biomass partitioning to grain yield formation. We hypothesized that (1) sulfur fertilization enhances maize yield primarily by regulating pre-silking sulfur and nitrogen acquisition and associated biomass accumulation; (2) sulfur-mediated nutrient–biomass regulatory processes are broadly conserved across contrasting soil types, while their magnitude is modulated by soil sulfur supply capacity; and (3) soil-specific differences in yield response to sulfur fertilization arise from variation in nutrient uptake efficiency and assimilate remobilization rather than from post-silking assimilation alone. This study provides process-based evidence for understanding how sulfur fertilization optimizes maize yield through coordinated regulation of nutrient uptake and biomass dynamics across contrasting soils, offering a scientific basis for soil-specific sulfur management in temperate spring maize systems of Northeast China.

## 2 Materials and methods

### 2.1 Experimental location and design

**Figure 1:**
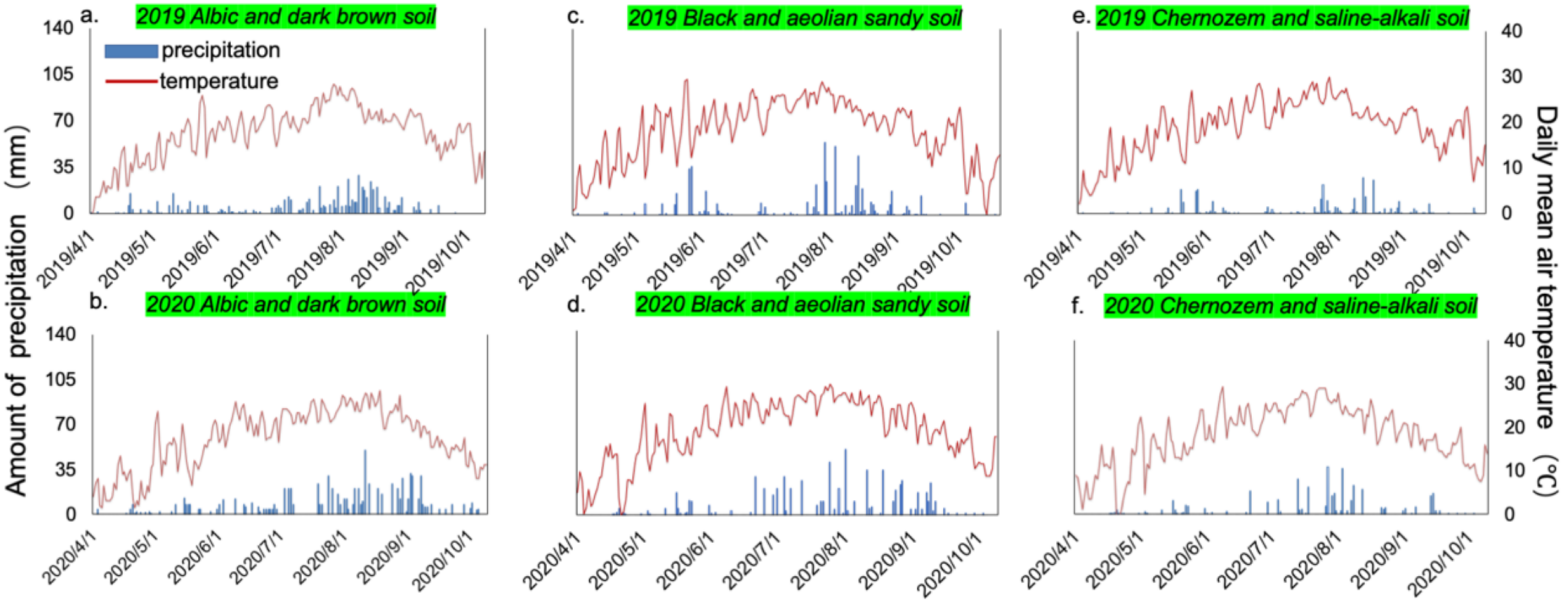
Among the six soil types, chernozem and saline-alkali soil are in Tongyu County, black soil and aeolian sandy soil are in Lishu County, and albic soil and dark brown soil in Longjing City, the study regions in the same city or county share a common set of mean daily temperature and precipitation data during the growth period.

The field trials were conducted across six distinct soil types in Northeast China, with the specific locations detailed in **Table 1**. These soil types include dark brown soil (42°47’ N, 129°20’ E), albic soil (42°46’ N, 129°19’ E), black soil (43°20’N, 124°4’E), aeolian sandy soil (44°35’ N, 123°7’ E), chernozem soil (43°21’ N, 124°5’ E), and saline-alkali (44°35’ N, 123°10’ E) soil. Climate and soil nutrient information for the trial fields is presented in **Table 1** and **Figure 1**. The experimental site featured the cultivation of the Liangyu 99 maize variety, which is a widely cultivated high-yielding maize hybrid in Northeast China and is representative of regional production systems. Drought stress was not observed in the reproductive phase. The precipitation conditions in Tongyu County during the two-year trial were consistent with the region’s semi-arid climate. These six soil types were selected to represent the major upland maize soils in Jilin Province, covering a wide range of inherent sulfur supply capacities and physicochemical properties.

**Table 1:** Trial field soil nutrient information (0–20 cm) tested before fertilizing in 2019. The precipitation data for 2019 and 2020 represents the cumulative rainfall from April, prior to planting, through October, following the harvest, for each respective year. Given the geographical proximity of the test sites, one precipitation dataset was utilized for both albic soil and dark brown soil, another dataset was employed for black soil and aeolian sandy soil, and a third dataset was shared for saline-alkali soil and chernozem soil.

Soil types	pH	Organic matter g kg^-1^	Available N mg kg^-1^	Olsen-P mg kg^-1^	Available K mg kg^-1^	Available S mg kg^-1^
Albic	5.79	19.8	103.8	15.7	152.3	16.4
Dark brown	5.68	22.6	113.6	15.8	170.6	15.4
Black	5.63	18.2	106.5	48.3	211.2	11.9
Aeolian sandy	6.77	11.3	61.7	24.9	160.9	10.6
Chernozem	7.98	15.6	88.7	15.7	163.7	10.1
Saline-alkali	8.43	10.2	35.6	8.6	200.1	10.2

### 2.2 Experimental design and management

During 2019-2020, two-year trials were conducted to determine maize grain yield, thousand-grain weight, and kernel number per ear at maturity stage (R6) on six types of soil. Fertilizer application rates were established in accordance with the practices of high-yielding maize farming in northeastern China, and these fertilizers were subsequently applied across all soil types in the field trials. Field trials on each soil type were designed to set up five sulfur nutrient levels of sulfur gradients with the same amount of N, P and K fertilizer inputs. All fertilizers, including sulfur-containing fertilizer, were applied as a single basal before sowing. Sowing was then carried out manually at a density of 65,000 ears ha^-1^. Each trial field was arranged in a randomized plot design. A randomized complete block design was used at each site, with sulfur treatments randomized within blocks to account for spatial heterogeneity. Each plot consisted of nine rows with a row spacing of 0.6 m and a length of 10 m. Nutrient inputs by treatment in field trials, no organic fertilizers were applied in the experiment, and a total of 5 kinds of chemical fertilizers were used. The nitrogen fertilizer used urea and control-releasing urea. Among them, the amount of control-releasing urea accounted for 20.45% of the total input of N, and diammonium phosphate was used for phosphate fertilizer. Potassium fertilizer uses potassium chloride; sulfur fertilizer used ammonium sulfate, and the specific dosage was shown in **Table 2**. Urea, controlled-release urea, ammonium sulfate, and diammonium phosphate collectively supplied a consistent total sum of nitrogen nutrients for every treatment. To avoid confounding effects between nitrogen and sulfur, total nitrogen input was kept constant across all treatments. Field weed management, using 24% nicosulfuron atrazine, was applied to all treatments 3 days after planting, and field insecticide management, using chlorpyrifos, was applied to all treatments 70 days after planting. Testing and sampling were conducted for different indicators throughout the reproductive period of maize growth.

**Table 2:** Application rates and plant density for each treatment.

Treatment code	Nitrogen N kg ha^-1^	Phosphorus P_2_O_5_ kg ha^-1^	Potassium K_2_O kg ha^-1^	Sulfur S kg ha^-1^	Seeding density ear·ha^-1^
S0	210	90	90	0	65000
S30	210	90	90	30	65000
S60	210	90	90	60	65000
S90	210	90	90	90	65000
S120	210	90	90	120	65000

### 2.3 Sampling and laboratory analyses

#### 2.3.1 Grain yield and its components

After the physiological maturity of the maize, 2 rows were eliminated from each side of the test plot, and 1 meter was removed from each end of the plot to avoid the sampling area during the trial. The remaining section of the plot served as the measuring area, and the effective area of this zone was determined. It was necessary to document the total ear count and combined ear weight within this measurement area, compute the average individual ear weight, and finally, weigh and choose 10 representative samples (with ears closely resembling the norm). The average single ear weight among these 10 samples equated to the average single ear weight within the measuring area for yield assessment. The maize ears were threshed after the yield measurement. A suitable number of maize kernels were weighed fresh, dried and weighed dry to calculate the moisture content of the maize kernels. The yield of maize in the test area was converted from the moisture content of the kernels to the final yield per unit (kg·ha^-1^ at 14% moisture content) in the field.

Before threshing the maize ears, we had to account for the number of kernels per ear. After threshing the maize ears, the kernels were dried in an oven at 60 °C. The kernels were mixed to a constant weight, and 1000 kernels were randomly selected, weighed and recorded.

#### 2.3.2 Biomass accumulation and nutrient (N and S) remobilization

In both growing seasons (2019–2020), three representative maize plants were selected from each plot at the silking stage (R1) and physiological maturity (R6). Each treatment consisted of three replicate plots, and three representative plants were sampled from each plot as biological replicates. Plants were separated into leaves, stems (including leaf sheaths, bracts, and cobs), and grains. All samples were oven-dried at 105 °C for 30 min followed by drying at 60 °C to a constant weight, and dry biomass of each organ was recorded.

Dried samples from each organ were homogenized, and subsamples (~50 g) were finely ground for nutrient analysis. Total sulfur (S) concentration was determined after acid digestion (HNO_3_–HClO_4_, 4:1 v/v) using inductively coupled plasma spectroscopy (ICP-OES; SHIMADZU ICPS-7500, Japan). Total nitrogen (N) concentration was quantified following H_2_SO_4_–H_2_O_2_ digestion using a Kjeldahl analyzer (KDY-9820, KETUO, China).

Organ-level N and S uptake (kg ha^-^¹) at R1 and R6 were calculated as:


N or S uptake=Dry biomass×N   or S concentration.

Nutrient remobilization and remobilization efficiency were calculated following standard methods analogous to sulfur:


$$\begin{array}{l} {\text{Nutrient remobilization (kg ha}}^{-1})\\ =\text{Nutrient in vegetative organs at R}1–\text{Nutrient in vegetative organs at R}6 \end{array}$$

$$\begin{array}{l} \text{Nutrient remobilization efficiency }\left(\%\right)\\ =\text{ Nutrient remobilization}/\text{Nutrient in vegetative organs at R}1\times 100 \end{array}$$

$$\begin{array}{l} \text{Contribution of nutrient remobilization to grain }\left(\%\right)\\ =\text{Nutrient remobilization}/\text{Grain nutrient content at R}6\times 100 \end{array}$$

These indices were calculated separately for N and S to quantify the contribution of pre-silking nutrient accumulation to grain nutrient formation.

### 2.4 Data analysis

All statistical analyses were performed using SPSS 18.0 (SPSS Inc., Chicago, IL, USA) and GraphPad Prism 9.0.0 for Mac (GraphPad Software, San Diego, CA, USA). Prior to analysis, data normality and homogeneity of variance were assessed using the Shapiro–Wilk and Levene tests, respectively. When necessary, data were log- or square root-transformed to meet the assumptions of analysis of variance (ANOVA). For physiological and agronomic traits, one-way or two-way ANOVA were conducted depending on the experimental design. Following a significant omnibus F-test, Tukey’s HSD test (or Tukey–Kramer for unequal sample sizes) was applied to compare treatment means while controlling the family-wise error rate. For multi-factorial experiments, a linear mixed-effects model was used with Year as a random effect and Soil type and S rate as fixed effects, including all two-way and three-way interactions. Ninety-five percent confidence intervals (95% CIs) were calculated and reported for treatment means and multiple comparisons. Logistic regression analyses were conducted in GraphPad Prism 9.0.0 to fit dose–response and growth curves. Path coefficient analysis followed the method of Dewey and Lu (1959) to evaluate the direct and indirect contributions of key physiological traits to yield formation. All graphical representations and curve fittings were generated using GraphPad Prism 9.0.0 for Mac.

## 3 Results

### 3.1 Yield response of maize to sulfur fertilization across soils, years and S rates

#### 3.1.1 Overall yield trends across soil types, years and sulfur gradients

**Figure 2:**
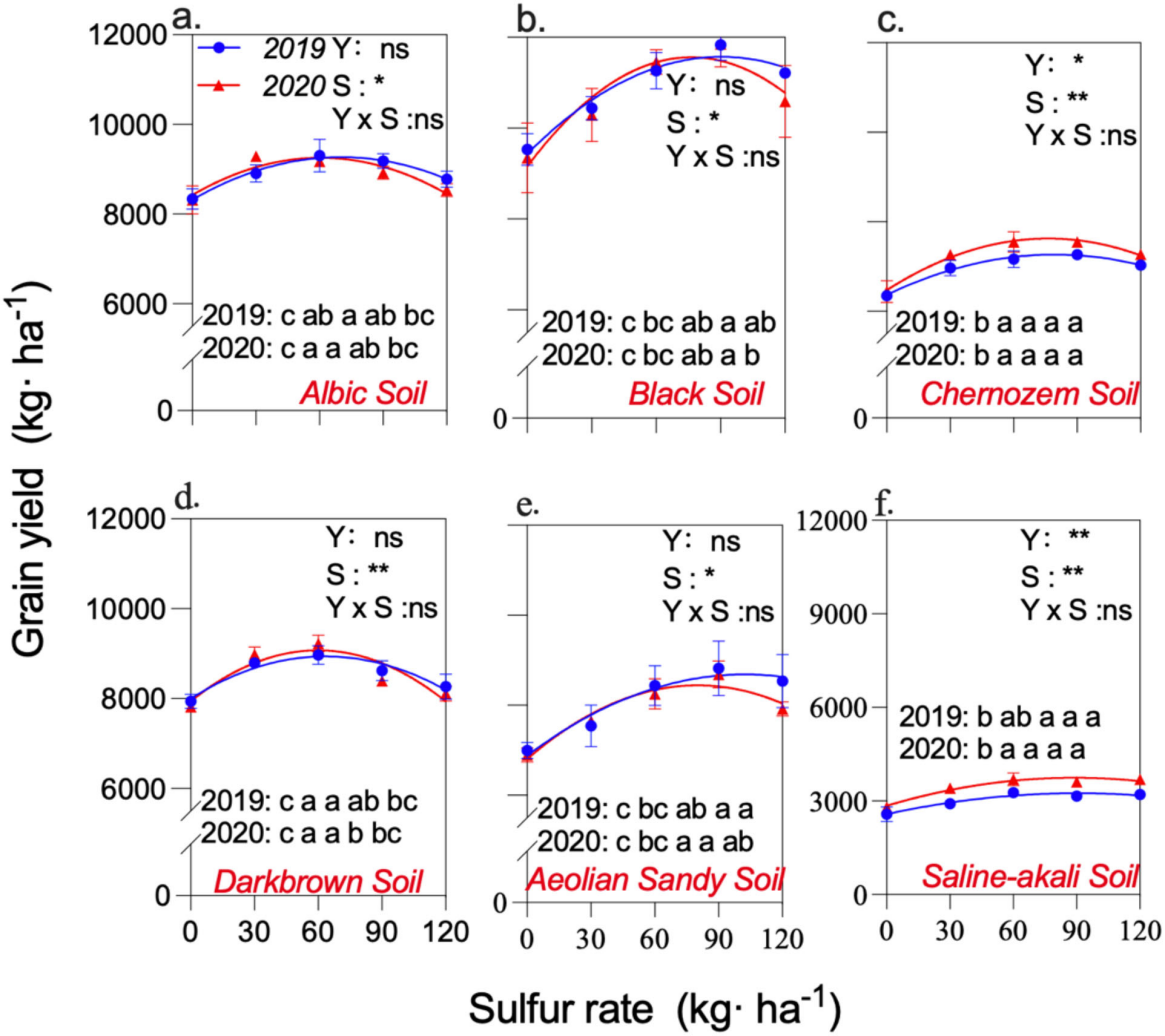
The figure illustrates the grain yield for the years 2019–2020 on various soil types, with each data point representing specific conditions. The curves represent the outcomes following regression analysis, where ‘Y’ denotes interannual factors, and ‘S’ signifies sulfur fertilizer input. The lowercase letters indicate the results of significance analysis for each sulfur application treatment within a given year. Error bars are given as S.E. NS and * indicate no significant and significant differences at P ≤ 0.05.

Maize grain yield differed significantly among the six soil types (**Figure 2**). Across the two years, the highest yield was observed in black soil (10,784 kg ha^-^¹ on average), followed by albic soil (8,861 kg ha^-^¹), dark brown soil (8,512 kg ha^-^¹), aeolian sandy soil (8,007 kg ha^-^¹), and chernozem soil (7,086 kg ha^-^¹), while the lowest yield occurred in saline–alkali soil (3,236 kg ha^-^¹).With increasing sulfur application, maize yield exhibited a general pattern of an initial increase followed by a decline in albic and dark brown soils. Two-year regression results showed that the average optimal S inputs in these soils were 66 kg ha^-^¹ and 58 kg ha^-^¹, producing yields of 9262 and 8903 kg ha^-^¹, respectively. In black soil and aeolian sandy soil, yield responses to sulfur were more pronounced, with the highest yields of 11,565 and 8,533 kg ha^-^¹ obtained at S inputs of 83 and 93 kg ha^-^¹, respectively. In chernozem soil and saline–alkali soil, the sulfur-induced yield response was weaker, and the maximum yields were 7447 and 3497 kg ha^-^¹, occurring at 78 and 90 kg S ha^-^¹, respectively. Across all soil types, the optimal S rates in 2020 were lower than those in 2019, with reductions of 2.0%–17.7%, indicating consistent year-to-year shifts in the S input required to achieve maximum yields.

#### 3.1.2 Nonlinear regression and soil-specific fitted optimum

Nonlinear regression analysis showed clear quadratic relationships between sulfur application rate and maize grain yield across the six soil types in both years (**Table 3**).

**Table 3:** Result of nonlinear regression of sulfur application and yield on six soil types, The table shows the equation, R^2^, the optimal sulfur input calculated from the equation and the optimal yield fitted to the optimal sulfur input from the quadratic nonlinear regression results for 2019 and 2020.

Soil types	Result of nonlinear regression		R^2^		Optimal S Inputs kg·ha^-1^		Optimal yield kg·ha^-1^	
	2019	2019	2019	2020	2019	2020	2019	2020
Albic soil	-0.1943x^2^+27.16x+8321	-0.227x^2^+27.47x+8424	0.74**	0.74**	70	61	9270	9255
Dark brown soil	-0.2201x^2^+27.44x+8025	-0.2617x^2^+28.48x+8152	0.70**	0.71**	62	54	8880	8926
Black soil	-0.2622x^2^+47.33x+9434	-0.4117x^2^+62.95x+9157	0.87**	0.69**	90	76	11569	11561
Aeolian sandy soil	-0.1725x^2^+35.28x+6881	-0.2338x^2^+39.23x+6736	0.72**	0.79**	102	84	8684	8381
Chernozem soil	-0.1428x^2^+22.69x+6366	-0.1983x^2^+30.28x+6471	0.89**	0.89**	79	76	7267	7626
Saline-alkali soil	-0.0815x^2^+14.8x+2578	-0.1136x^2^+20.21x+2847	0.80**	0.84**	91	89	3249	3745

* Represents p< 0.05 for R2 > 0.232, and ** implies p< 0.01 for R2 > 0.367 (n = 15).

For albic soil, the yield–S relationship was fitted by y = −0.1943x² + 27.16x + 8321 (2019) and y = −0.2270x² + 27.47x + 8424 (2020), both with R² = 0.74, indicating optimal S rates of 70 and 61 kg ha^-^¹ and corresponding optimal yields of 9270 and 9255 kg ha^-^¹.In dark brown soil, the regression models produced R² values of 0.70 and 0.71, giving optimal S rates of 62 and 54 kg ha^-^¹, associated with yields of 8880 and 8926 kg ha^-^¹.In black soil, the regression fits showed higher explanatory power (R² = 0.87 and 0.69), and the optimal S rates were 90 and 76 kg ha^-^¹, generating optimal yields of 11,569 and 11,561 kg ha^-^¹.For aeolian sandy soil, the regression models yielded R² values of 0.72 and 0.79, with optimal S inputs of 102 and 84 kg ha^-^¹ and optimal yields of 8684 and 8381 kg ha^-^¹.In chernozem soil, both years showed strong regression fits (R² = 0.89 in both years), with optimal S rates of 79 and 76 kg ha^-^¹, corresponding to yields of 7267 and 7626 kg ha^-^¹.In saline–alkali soil, R² values of 0.80 and 0.84 were obtained, and the optimal S rates were 91 and 89 kg ha^-^¹, producing optimal yields of 3249 and 3745 kg ha^-^¹.

Across all soils and years, the optimal sulfur application rates ranged from 54 to 102 kg ha^-^¹, depending on soil type and production year.

### 3.2 Response of maize yield components to sulfur fertilization

Since sulfur fertilization significantly altered grain yield, we next examined how the individual yield components responded to S inputs across soil types. Maize thousand-kernel weight (TKW) and kernel number per ear (GN) showed significant differences in their responses to sulfur fertilization across the six soil types. Based on two-year data from all soils, nonlinear regression using quadratic equations was performed for GY, TKW, and GN. All regressions were statistically significant (p< 0.05), indicating that the fitted curves effectively captured the response patterns.

**Figure 3:**
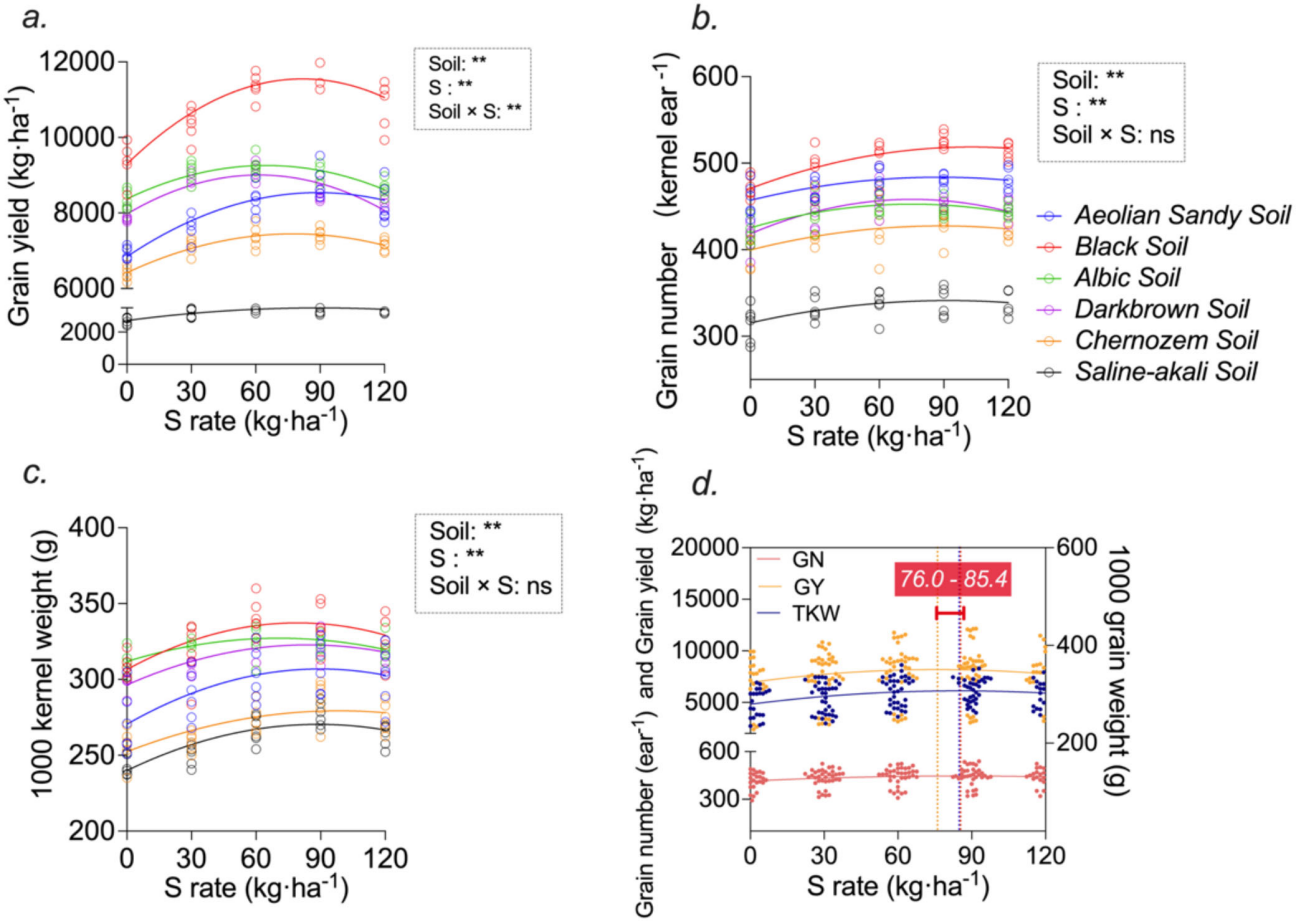
Data points for the regression analysis in **(a-c)** are for each soil type corresponding to all replicates of treatment. The factor Year is regarded as a repeating factor (n=30), and the middle indicator in d is the regression analysis for all field data between the two years (n=180).

As shown in **Figures 3a, c**, both grain yield (GY) and TKW increased with sulfur application and then declined at higher S rates in all six soil types. The response of GN, illustrated in **Figure 3b**, also followed an increasing–decreasing trend, although the curvature was weaker than that observed for GY and TKW. Separate nonlinear regressions for each soil type in both years yielded significant fits for all three yield components.

Across soils and years, the optimal sulfur application range derived from the regression curves was 76.0–85.4 kg S ha^-^¹ (**Figure 3d**). Within this range, sulfur application increased grain yield by 11.4–29.4%, enhanced thousand-kernel weight by 22–37.6 g, and raised kernel number per ear by 5.2–11.4% over the unfertilized control.

### 3.3 Path coefficients of maize yield components across six soil types

To further quantify the relative contributions of yield components to final grain yield, we performed a path analysis across the six soil types. Path analysis showed that, except in saline–alkali soil, ear number per hectare (EN) had no significant effect on grain yield (GY) in the other five soil types. In contrast, kernel number per ear (GN) and thousand-kernel weight (TKW) exhibited significant effects on GY across all soils. In saline–alkali soil, EN was significantly correlated with GN (p< 0.01), TKW (p< 0.05), and GY (p< 0.01), whereas no significant correlations were detected for EN in the remaining soils.

The contributions of GN and TKW to grain yield varied among soil types. For example, the path coefficient of GN on GY was 0.656 (p< 0.01) in black soil and 0.362 (p< 0.05) in albic soil. The contribution of TKW to GY was highest in aeolian sandy soil (0.444, p< 0.01) and lowest in albic soil (0.125, p< 0.01). Across all six soils, both GN and TKW showed significant positive contributions to grain yield.

### 3.4 Sulfur accumulation and remobilization

Because yield responses are physiologically driven by nutrient acquisition, we evaluated sulfur uptake, translocation and remobilization. Table unveils the disparity in pre-silking sulfur accumulation during the vegetative growth phase and post-silking sulfur remobilization during the reproductive growth phase in maize, influenced by three key factors: soil type, year, and sulfur application. The contribution of remobilization sulfur to grain was 48.3% in two years. Moreover, among the six soil types, sulfur accumulation in the R1 and maturity stages of maize on black soil was significantly higher than that in aeolian sandy soil, albic soil, dark brown soil and chernozem soil in that order. On the other hand, saline-alkali soil was the lowest, and in addition to sulfur remobilization, black soil donated the highest remobilization efficiency for which 1.8-11.7 percentage points outweighed the other soil types under experimental conditions. Sulfur significantly enhanced 53-106% maize sulfur accumulation at R1 and 41-171% at maturity, compared to the no-sulfur treatment. Besides, the elevation of pre-silking sulfur accumulation as a percentage of total sulfur at maturity (from 68% at S30 to 74% at S120), as well as increased sulfur remobilization in straw after-silking. Notably, the sulfur remobilization efficiency progressively improved with the amount of sulfur applied in maize and simultaneously increased the sulfur amounts and proportions within the straw remobilize to the kernel. In addition, sulfur facilitated 11.5-15.9 percentage points of remobilization efficiency of pre-silking sulfur accumulation to grain, ultimately improving the contribution of remobilized sulfur to grain.

### 3.5 Nitrogen accumulation and remobilization

Given the strong biochemical coupling between sulfur and nitrogen metabolism, we next assessed how S fertilization influenced nitrogen uptake and remobilization. Sulfur fertilization significantly influenced maize nitrogen (N) uptake, N distribution at maturity, and N remobilization across the six soil types examined (**Table 4**). Total N accumulation at maturity differed markedly among soils, ranging from 92.5 kg ha^-^¹ in saline–alkali soil to 216.6 kg ha^-^¹ in black soil. Across all soils, sulfur application increased total N uptake relative to S0, with the largest increments observed in black and aeolian sandy soils (≈40–80 kg ha^-^¹ across S rates) and the smallest increments in saline–alkali soil (≈20 kg ha^-^¹). Grain N accounted for 55–65% of total plant N across soils. Both straw N and grain N increased progressively from S0 to S60–S90, with no further increase detected at S120.

**Table 4:** N accumulation and remobilization of maize under different sulfur inputs on six soil types during 2019-2020.

Soil type	N accumulation at R1 kg ha^-1^	N accumulation at maturity kg ha^-1^			Remobilization of N kg ha^-1^	N remobilization efficiency %	Contribution to grain by N remobilization %
		Straw	Grain	Total			
Albic soil	118.4b	68.9bc	103.0bc	171.9bc	49.5bc	41.8b	48.1ab
Dark brown soil	121.7b	71.2b	107.2b	178.4b	50.5b	41.5b	47.1b
Black soil	140.8a	78.2a	138.4a	216.6a	62.6a	44.5a	45.2b
Aeolian sandy soil	125.9ab	78.6a	109.0b	187.6ab	47.3bc	37.6c	43.4b
Chernozem soil	108.5c	63.3c	91.2c	154.5c	45.2c	41.7b	49.5a
Saline-alkali soil	62.1d	41.5d	51.0d	92.5d	20.6d	33.2d	40.4c
Year							
2019	117.4a	69.4a	104.0a	173.4a	48.0a	40.9a	46.1a
2020	120.2a	70.2a	105.7a	175.9a	50.0a	41.6a	47.0a
S rate							
S0	90.1c	56.4c	84.8c	141.2c	33.7c	37.4d	39.7c
S30	105.0b	63.2b	94.5b	157.7b	41.8b	39.8c	44.2b
S60	117.7ab	69.1ab	103.3ab	172.4ab	48.6ab	41.3ab	47.0ab
S90	128.6a	74.0a	111.0a	185.0a	54.6a	42.5a	49.0a
S120	122.4a	71.0a	106.8a	177.8a	51.4a	42.0a	48.1ab
ANVOA							
Soil	**	**	**	**	**	*	*
Year	ns	ns	ns	ns	*	ns	ns
S rate	**	**	**	**	**	**	**
Soil × Year	ns	ns	ns	ns	ns	ns	ns
Soil × S	*	**	**	**	**	**	*
Year × S	ns	ns	ns	*	ns	ns	ns
Soil × Year × S	ns	ns	ns	ns	ns	ns	ns

Values followed by different letters within the same factor are significantly different at P ≤ 0.05. * Represents p< 0.05, and ** implies p< 0.01.

Nitrogen remobilization from vegetative organs ranged from 20.6 to 62.6 kg ha^-^¹ across soil types and sulfur treatments, with black soil showing the highest values and saline–alkali soil the lowest. Sulfur fertilization increased N remobilization compared with S0, and the highest remobilization values were generally observed under S60 and S90. Nitrogen remobilization efficiency ranged from 33.2% to 44.5%, with the highest efficiencies occurring in black soil and under S60–S90.The contribution of N remobilization to grain N ranged from 39.7% to 50.0%, with the highest values in chernozem and albic soils. Sulfur application increased the contribution of remobilized N relative to S0, with an improvement of 6–10 percentage points at S60–S90. Across soils, the proportion of grain N derived from remobilization remained lowest in saline–alkali soil (<41%) and highest in more fertile soils (>47%). Year effects were non-significant for most traits, whereas the effects of S rate and the Soil × S interaction were consistently significant (**Table 4**). The S60–S90 range produced the highest values for total N uptake, vegetative N storage, and N remobilization across soils.

### 3.6 Sulfur (S%), nitrogen (N%) concentrations and N/S ratios at silking and maturity

Marked differences in sulfur and nitrogen concentrations were observed among soil types and S fertilization regimes at both R1 and R6 (**Table 5**). Sulfur concentrations at R1 remained relatively narrow across soils (0.12–0.14%), but pronounced divergence emerged at maturity, with grain S consistently exceeding straw S, reflecting the preferential allocation of sulfur to metabolically active sink tissues. Nitrogen concentration displayed an even stronger gradient between organs, as grain N reached 1.35–1.83%, more than double the straw N concentrations.

**Table 5:** Sulfur (S%) and nitrogen (N%) concentrations at silking and maturity, and N/S ratios across soil types, years, and sulfur rates.

Soil type	R1	R6		R1	R6		N/S ratio at R1	N/S ratio at R6
	S (%)	S in straw (%)	S in grain (%)	N at R1 (%)	N in straw (%)	N in grain (%)		
Albic soil	0.12b	0.08b	0.14b	1.08b	0.70b	1.35c	8.7a	9.1a
Dark brown soil	0.13a	0.09b	0.14b	1.13a	0.71b	1.46b	8.7a	9.3a
Black soil	0.12b	0.08b	0.16a	1.12a	0.74b	1.49b	9.1a	9.1a
Aeolian sandy soil	0.14a	0.10a	0.17a	1.22a	0.87a	1.59b	9.1a	9.2a
Chernozem soil	0.13a	0.08b	0.15a	1.13a	0.69b	1.50b	8.8a	9.3a
Saline-alkali soil	0.12b	0.08b	0.15a	0.93b	0.63b	1.83a	8.1b	9.4a
2019	0.13a	0.09a	0.15b	1.17a	0.76a	1.57a	9.3a	9.7a
2020	0.13a	0.09a	0.16a	1.17a	0.76a	1.58a	9.2a	9.5b
S0	0.08c	0.07c	0.11d	0.99a	0.65b	1.42a	11.9a	11.8a
S30	0.12b	0.08a	0.14c	1.07a	0.71a	1.43a	9.0b	9.3b
S60	0.13b	0.09b	0.16b	1.10a	0.73a	1.47a	8.5b	8.7b
S90	0.14a	0.09a	0.17b	1.21a	0.79a	1.58a	8.5c	9.0b
S120	0.15a	0.10a	0.18a	1.16a	0.74a	1.60a	7.8c	8.4b

Values are means; different lowercase letters indicate significant differences (p< 0.05).

The resulting N/S stoichiometric patterns revealed clear S-mediated adjustments in nutrient balance. Plants under S deficiency (S0) exhibited elevated N/S ratios (>11), indicating constrained sulfur supply relative to nitrogen assimilation. In contrast, moderate S inputs (S60–S90) maintained N/S ratios near 8–9, a range commonly associated with optimal enzymatic and protein synthesis capacity in maize. Across soil types, the lowest N/S ratios corresponded with soils exhibiting the strongest S responsiveness, suggesting a direct link between S availability, nutrient balancing, and physiological efficiency during grain filling.

### 3.7 Biomass allocation, remobilization, and harvest index as affected by sulfur fertilization

Since nutrient accumulation ultimately affects biomass production, we analyzed biomass allocation patterns and remobilization efficiency. Sulfur fertilization significantly influenced biomass allocation before and after silking across soil types, years, and S rates. Pre-silking biomass accounted for 62–65% of total aboveground biomass in Albic, dark brown, black, aeolian sandy, and chernozem soils, and exceeded 70% in the saline–alkali soil. Year effects were not significant for pre- or post-silking biomass proportions.

Across S rates, the proportion of pre-silking biomass increased slightly from 62.4% at S0 to 63.3–64.9% at S30–S90 and remained similar at S120 (64.7%). Correspondingly, biomass remobilization increased from 461 kg ha^-^¹ at S0 to 918–1249 kg ha^-^¹ at S30–S90. Remobilization efficiency increased from 5.0% at S0 to 9.3–11.7% at S30–S90, and the contribution of remobilized biomass to grain increased from 7.7% to 13.8–17.8% across the same range of S treatments.

Harvest index (HI) differed significantly among soils and S rates. HI ranged from 39.9% to 46.8% across soil types and was lowest in the saline–alkali soil (29.7%). Across S rates, HI increased from 40.8% at S0 to 42.6–42.7% at S30–S90, followed by a slight decrease at S120 (41.1%). Year had no significant effect on HI, while Soil × S interactions were significant for most biomass variables.

### 3.8 Relationship between pre- and post-silking biomass and grain yield of maize

**Figure 4:**
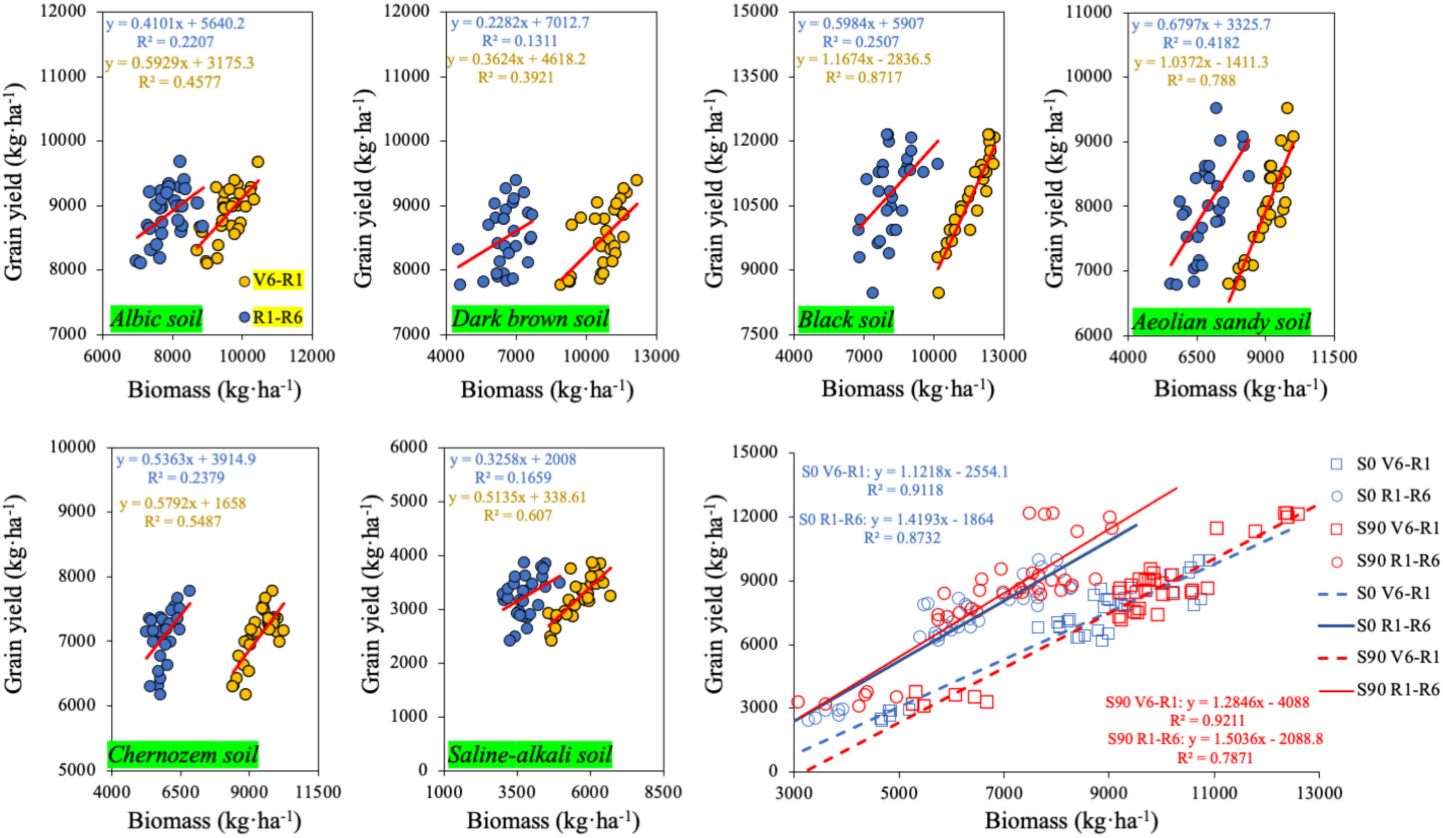
Relationship between pre- and post-flowering biomass and corn yield on six soil types.

To clarify the physiological basis of S-induced yield improvement, we examined the relationships between pre- and post-silking biomass accumulation and grain yield. According to the relationships shown in **Figure 4**, biomass accumulation during both the pre-silking period (V6–R1) and post-silking period (R1–R6) was positively correlated with grain yield across all soil types. In all treatments, the correlation coefficients between pre-silking biomass (V6–R1) and grain yield were higher than those between post-silking biomass (R1–R6) and grain yield.

The strength of the relationships varied among soils. Black soil showed the highest correlation between pre-silking biomass and grain yield (R² = 0.87), while aeolian sandy soil exhibited the highest correlation between post-silking biomass and grain yield (R² = 0.41). Saline–alkali soil had the lowest pre-silking biomass accumulation and the weakest correlations with yield for both periods.

Across all six soil types, the S90 treatment consistently produced the highest biomass accumulation and the highest grain yield. Comparisons between S0 and S90 across soils showed that sulfur application increased the contribution of pre-silking biomass to grain yield more strongly than that of post-silking biomass.

## 4 Discussion

### 4.1 Sulfur-mediated nutrient regulation as a driver of yield improvement across soils

In this paper, we demonstrate that sulfur fertilization systematically improved maize performance across six contrasting soil types by coordinating nutrient balance, canopy development, and biomass allocation. This pattern is clearly reflected in the yield responses across S rates (**Figure 2**) and the nonlinear regression–derived optimal S levels (**Table 3**). The overall trend agrees with global evidence that S has re-emerged as a limiting nutrient due to declining atmospheric deposition and the widespread use of S-free fertilizers (Aula et al., 2019). Earlier studies reported that sulfur enhances leaf function, redox homeostasis, and N assimilation efficiency in cereals (Koprivova et al., 2000; Kopriva, 2004; Liu et al., 2020), and our results corroborate these findings.

Importantly, our dataset reveals strong soil-dependent differentiation in S responsiveness: black soil and aeolian sandy soil showed pronounced improvements in both yield components (**Figure 3**) and nutrient acquisition (**Tables 4**, **6**), whereas saline–alkali soil exhibited minimal gains. Such differences suggest that S limitation interacts strongly with soil physicochemical constraints (Bejandi et al., 2009; Kulczycki, 2021; Afsar et al., 2017). Previous studies often report uniform crop–S response curves (A. et al., 2013; Balpande et al., 2016; Kulczycki, 2021), but our six-soil comparison demonstrates that spatial variation in soil fertility and stress tolerance must be accounted for in S management.

**Table 6:** Sulfur accumulation and remobilization of maize under different sulfur inputs on six soil types during 2019-2020.

Soil type	Sulfur accumulation at R1 kg ha^-1^	Sulfur accumulation at maturity kg ha^-1^			Remobilization of sulfur kg ha^-1^	Sulfur remobilization efficiency %	Contribution to grain by sulfur remobilization %
		Straw	Grain	Total			
Albic Soil	13.6b	8.1bc	10.7bc	18.8bc	5.5ab	40.7a	51.7a
Dark brown soil	14.0b	8.7a	10.5bc	19.2b	5.4ab	38.1b	50.8ab
Black soil	15.4a	8.9a	14.9a	23.8a	6.5a	42.5a	43.9b
Sandy soil	13.9b	8.7a	11.7b	20.4b	5.2ab	37.6b	44.4b
Chernozem Soil	12.3c	7.6b	9.0c	16.6c	4.7b	38.2b	52.2a
Saline-alkali soil	7.7d	5.5c	4.3d	9.9d	2.2c	28.8c	51.3a
Year							
2019	12.6a	7.8a	10.0a	17.8b	4.9a	38.5a	48.7a
2020	13.0a	8.1a	10.4a	18.5a	5.0a	38.3a	47.9a
S rate							
S0	7.6c	5.7c	6.3c	12.0c	1.9c	25.5b	30.7c
S30	11.7b	7.4b	9.6b	17.0b	4.3b	37.0a	44.8b
S60	13.9b	8.4a	11.4ab	19.8a	5.5a	39.5a	48.4b
S90	15.2a	8.8a	11.8a	20.6a	6.4a	41.9a	54.1a
S120	15.7a	9.2a	12.0a	21.2a	6.5a	41.4a	54.5a
ANVOA							
Soil	**	*	**	**	*	*	*
Year	ns	ns	ns	*	ns	ns	ns
S rate	**	**	**	**	**	*	**
Soil × Year	ns	ns	ns	ns	ns	ns	ns
Soil × S	*	**	**	**	**	**	**
Year × S	ns	ns	ns	*	Ns	ns	ns
Soil × Year × S	ns	ns	ns	ns	Ns	ns	ns

Values followed by different letters within the same factor are significantly different at P ≤ 0.05. * Represents p< 0.05, and ** implies p< 0.01.

Maize yield formation is particularly sensitive to assimilate and nutrient supply during the critical period surrounding silking, as pre-silking growth determines floret survival and kernel set, making kernel number especially responsive to nutrient availability before silking (Song et al., 2024; Shen et al., 2025). Consistent with this framework, we observed that the beneficial effects of sulfur fertilization were largely expressed before silking. This pattern agrees with previous studies showing that pre-silking biomass accumulation is the dominant determinant of kernel number in maize (Salvagiotti et al., 2009; Bonelli et al., 2016). This is supported by our strong relationships between early biomass and yield (**Figure 4**) and by the increased N/S ratios at silking (**Table 5**). While nitrogen has been recognized as the primary driver of source formation (Ciampitti and Vyn, 2012), few studies have evaluated sulfur’s parallel role. Our findings demonstrate that S fertilization improved N/S stoichiometry and accelerated N uptake during the critical period of ear formation, consistent with biochemical evidence that S is required for the synthesis of cysteine, methionine, and N-assimilation enzymes (Carciochi et al., 2017, Carciochi et al., 2020; Department of Agronomy et al., 2017). Collectively, this study fills an important knowledge gap by quantifying multi-soil consistency in S-driven early vigor and nutrient coordination.

### 4.2 Biomass accumulation and remobilization as central mechanisms underlying sulfur-induced yield gains

The improvements in N/S balance under moderate S supply likely underpinned the enhanced pre-silking growth, aligning with physiological studies demonstrating that balanced S availability promotes protein synthesis, chlorophyll stability, and glutathione-based redox protection (Fatma et al., 2014, Fatma et al., 2021; Ahmad et al., 2016). In our study, S application-maintained N/S ratios within the optimal physiological range (**Table 5**), enabling rapid N uptake and greater biomass accumulation at R1. This supports earlier suggestions that sulfur functions as a co-limiting factor for N assimilation in high-yield maize systems (Byers and Bolton, 1979; A. et al., 2013).

Enhanced biomass remobilization under S60–S90 (**Table 7**) further indicates that sulfur strengthens source–sink coordination, consistent with principles that early biomass sets the ceiling for storage remobilization capacity (Edmeades and Daynard, 1979; Tsimba et al., 2013). This is mirrored by increases in remobilized S and N (**Tables 4**, **6**), suggesting that sulfur not only improves assimilation but also facilitates later redistribution of nutrients to the grain.

**Table 7:** Biomass accumulation and remobilization of maize under different sulfur inputs on six soil types during 2019-2020.

Treatment	Biomass at silking kg ha^−1^	Stalk & leaf at maturity kg ha^−1^	Grain at maturity kg ha^−1^	Harvest index %	Biomass remobilization kg ha^−1^	Remobilization efficiency %	Contribution to grain by remobilization %
Albic soil	11000 b	9866 b	7632 b	43.6 b	1134 b	10.3 bc	14.9 ab
Dark brown soil	10744 b	9993 b	7320 c	42.3 b	751 c	7.0 cd	10.3 bc
Black soil	12617 a	10550 a	9274 a	46.8 a	2067 a	16.4 a	22.3 a
Aeolian sandy soil	10298 c	9014 c	6857 d	43.2 b	1284 b	12.5 bc	18.7 a
Chernozem soil	9616 c	9192 c	6094 e	39.9 c	424 cd	4.4 de	7.0 cd
Saline–alkali soil	6689 d	6595 d	2783 f	29.7 d	94 d	1.4 e	3.4 d
2019	10060 a	9116 a	6640 a	42.1 a	944 a	9.4 a	14.2 a
2020	10261 a	9287 a	6680 a	41.8 a	974 a	9.5 a	14.6 a
S0	9140 b	8678 b	5975 b	40.8 c	461 c	5.0 b	7.7 b
S30	9829 ab	8911 ab	6625 a	42.6 b	918 b	9.3 a	13.8 a
S60	10670 ab	9438 a	7007 a	42.6 ab	1233 b	11.6 a	17.6 a
S90	10648 a	9399 a	7017 a	42.7 a	1249 a	11.7 a	17.8 a
S120	10516 a	9581 a	6676 a	41.1 bc	934 a	8.9 a	14.0 a
ANOVA							
Soil	**	**	**	**	**	**	**
Year	ns	Ns	ns	*	ns	ns	ns
S rate	**	**	**	**	**	**	**
Soil × Year	ns	Ns	ns	ns	ns	ns	ns
Soil × S	*	*	**	*	**	*	*
Year × S	ns	Ns	*	ns	ns	ns	ns
Soil×Year × S	ns	Ns	ns	ns	ns	ns	ns

Values followed by different letters within the same factor are significantly different at P ≤ 0.05. * Represents p< 0.05, and ** implies p< 0.01.

**Figure 5:**
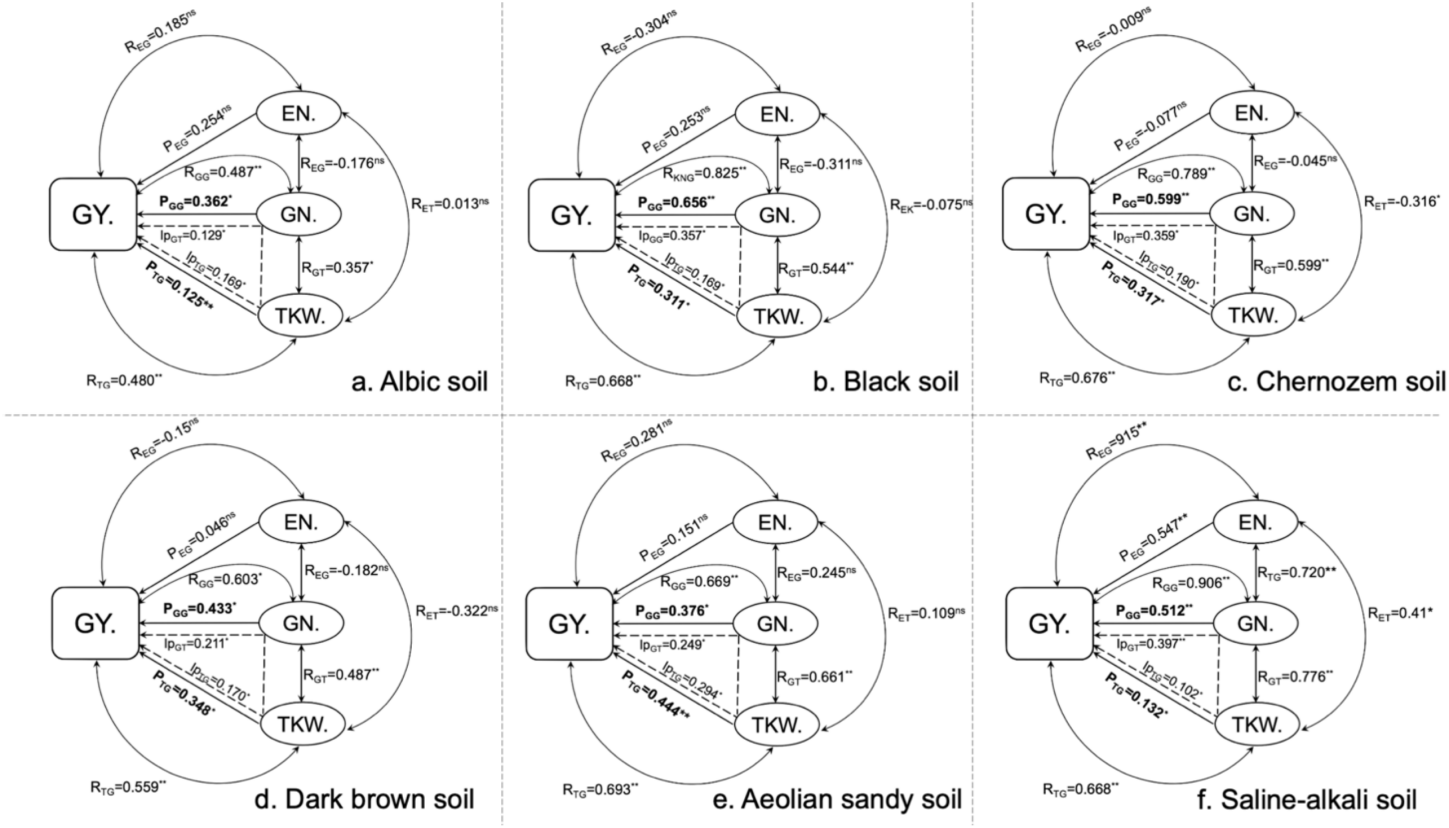
Diagram **(a–f)** shows the correlation coefficients and path coefficients of yield components affecting grain yield. The correlation coefficients are represented by the double-arrowed line with R. The line with a single arrow indicates the direct path coefficient donated with P, and the short line with a single arrow indicates the indirect path coefficient. IpTG and IpGT refer to the indirect path coefficients from TKW through GN to grain yield, and from GN through TKW to grain yield, respectively. * and ** represent significant differences at the 0.05 and 0.01 levels.

Kernel number emerged as the strongest determinant of yield across soils (**Figure 5**), consistent with both crop physiology theory and genetic evidence (Bonelli et al., 2016; Kitonyo et al., 2018; Jia et al., 2020). The absence of further yield improvement under S120—despite higher S uptake (**Table 6**)—supports a saturation effect, in which sink capacity rather than nutrient availability becomes the limiting factor once early-season processes are optimized. This complements earlier findings that excessive S does not enhance photosynthesis or grain filling beyond a physiological optimum (Ashida et al., 2005; Mehmood et al., 2021).

Taken together, the integrated evidence from yield components (**Figure 3**), path analysis (**Figure 5**), biomass allocation (**Table 7**), and biomass dynamics (**Figure 4**) demonstrates that sulfur enhances maize productivity primarily by increasing early assimilate generation and strengthening structural development of the ear, rather than by late-season carbon provision alone.

### 4.3 Soil-dependent sulfur responsiveness and implications for optimizing S inputs

From a management perspective, it is important to distinguish between soil-specific optimal sulfur rates derived from individual yield response fittings and the narrower sulfur range discussed here as an integrated reference. In the Results section, optimal sulfur application rates varied among soils and years (54–102 kg S ha^-^¹), reflecting differences in inherent sulfur supply capacity and soil constraints. By contrast, the range of approximately 76–85 kg S ha^-^¹ discussed here represents a synthesis based on combined evidence from yield components, nutrient uptake, biomass accumulation, and remobilization across soils under the studied conditions, rather than a universal optimum.

This interpretation is consistent with large-scale agronomic surveys showing that crops frequently respond to sulfur fertilization, with yield increases of 7–30% reported at 87% of 572 field sites worldwide (Hu et al., 2002), indicating a broad but not uniform sulfur demand across environments. In maize-specific studies, sulfur application at approximately 80 kg S ha^-^¹ has been reported to improve plant height, biomass production, ear development, kernel number per ear, and grain weight, while simultaneously enhancing grain protein quality through increased accumulation of sulfur-containing storage proteins (Liu et al., 2021); Nanthakumar et al., 2014.). Together with multi-site cereal studies reporting similarly narrow intermediate sulfur ranges when yield formation is evaluated alongside nutrient and biomass traits (Kulhánek et al., 2011; Kovar, 2021; Gautam and Mishra, 2025), these findings support the use of an intermediate sulfur range as a contextual reference rather than a fixed recommendation.

Nevertheless, the magnitude of yield improvement differed substantially among soils in the present study, mirroring variations in sulfur accumulation, nitrogen uptake, biomass remobilization efficiency, and N/S homeostasis. This highlights that sulfur fertilization responses are strongly modulated by soil-specific constraints such as salinity, organic matter deficiency, or restricted rooting depth (Tong et al., 2014; Carciochi et al., 2017). Accordingly, our results support soil-classified sulfur management strategies, in which soil-specific optimal rates derived from local response curves remain the primary basis for decision-making, while integrated sulfur ranges provide a comparative reference across sites and studies.

### 4.4 Limitations and future directions

Although this study includes six representative soils, the findings may still be influenced by the cool-temperate climate of Northeast China. In warmer or water-limited systems, the relative contribution of post-silking photosynthesis may shift, potentially altering S-mediated improvements (Wang et al., 2020, Wang et al., 2023). Genotypic variation in sulfur-use efficiency was not evaluated, despite evidence that hybrids differ in S sensitivity (Friedrich and Schrader, 1978; Kaya et al., 2020). Soil microbial S mineralization was not measured and may partly explain soil-specific responsiveness.

Future research should investigate:

- N–S temporal coordination via split or localized placement.
- genotype × S interactions to identify S-efficient cultivars.
- root and rhizosphere processes underlying S-enhanced nutrient uptake.
- long-term soil sulfur balances to evaluate sustainability
- integration of S fertilization into precision agriculture frameworks for site-specific recommendations.

Importantly, the enhanced N uptake under sulfur fertilization (**Table 4**) confirms the biochemical interdependence between the two nutrients. This suggests that sulfur management could be incorporated into regional nutrient strategies to optimize N use efficiency and stabilize yield formation under variable soil fertility.

## 5 Conclusions

This work shows that sulfur fertilization offers a practical and robust strategy to improve maize productivity across diverse soil environments. By enhancing early-season nutrient capture and biomass accumulation, sulfur strengthens the physiological foundation for yield formation and improves the efficiency of assimilate use during grain filling. The consistent identification of a moderate optimal S range (75–90 kg ha^-^¹) provides an actionable guideline for regional fertilization programs, reducing the risk of under- or over-application. Soil-dependent responsiveness further highlights the need to integrate sulfur with broader soil fertility management, especially in high-yielding or nutrient-responsive soils. Overall, the results support the adoption of sulfur fertilization as a key agronomic lever for sustaining maize yields and nutrient-use efficiency in Northeast China.

## Data Availability

The raw data supporting the conclusions of this article will be made available by the authors, without undue reservation.
